# Horizontal Flow Constructed Wetland for Greywater Treatment and Reuse: An Experimental Case

**DOI:** 10.3390/ijerph17072317

**Published:** 2020-03-30

**Authors:** Maria Cristina Collivignarelli, Marco Carnevale Miino, Franco Hernan Gomez, Vincenzo Torretta, Elena Cristina Rada, Sabrina Sorlini

**Affiliations:** 1 Department of Civil Engineering and Architecture, University of Pavia, via Ferrata 1, 27100 Pavia, Italy; mcristina.collivignarelli@unipv.it (M.C.C.); marco.carnevalemiino01@universitadipavia.it (M.C.M.); 2 Interdepartmental Centre for Water Research, University of Pavia, Via Ferrata 3, 27100 Pavia, Italy; 3 Department of Civil, Environmental, Architectural Engineering and Mathematics, University of Brescia, Via Branze 43, 25123 Brescia, Italy; franco.gomez@unibs.it (F.H.G.); sabrina.sorlini@unibs.it (S.S.); 4 Department of Theoretical and Applied Sciences, University of Insubria, Via G.B. Vico 46, 21100 Varese, Italy; vincenzo.torretta@uninsubria.it

**Keywords:** circular economy, constructed wetland, *Escherichia coli*, greywater, macrophytes, reuse, resource recovery, wastewater, water scarcity

## Abstract

In the coming years, water stress is destined to worsen considering that the consumption of water is expected to increase significantly, and climate change is expected to become more evident. Greywater (GW) has been studied as an alternative water source in arid and semiarid zones. Although there is no single optimal solution in order to treat GW, constructed wetlands proved to be effective. In this paper, the results of the treatment of a real GW by a horizontal flow constructed wetland (HFCW) for more than four months are shown. In the preliminary laboratory-scale plant, *Phragmites australis*, *Carex oshimensis* and *Cyperus papyrus* were tested separately and showed very similar results. In the second phase, pilot-scale tests were conducted to confirm the performance at a larger scale and evaluate the influence of hydraulic retention time, obtaining very high removal yields on turbidity (>92%), total suspended solids (TSS) (>85%), chemical oxygen demand (COD) (>89%), and five-day biological oxygen demand (BOD_5_) (>88%). Based on the results of the pilot-scale HFCW, a comparison with international recommendations by World Health Organization and European Union is discussed.

## 1. Introduction

Water stress is now a reality in many parts of the world [1,2,3,4]. This phenomenon is destined to worsen considering that the consumption of water is expected to increase significantly in the coming years and with now more evident climate change accentuating this phenomenon [5]. In the European Union (EU), at least 17% of the territory and 11% of the population is affected by water scarcity [6,7,8,9,10,11]. In the Mediterranean area, over 20% of the population lives under constant water stress and in the summer this percentage reaches 50% as defined by the European Environmental Agency’s (EEA) water exploitation index (WEI) [12,13,14].

In addition to reducing water consumption, a possible solution can be the reuse of treated wastewater (WW) produced by human activities [15,16,17]. The WW presents a very large number of contaminants such as dyes, surfactants, heavy metals, drugs, personal care products and bacteria [18,19,20,21,22,23]. To date, there are numerous treatments to remove the contaminants present in the WW, but there is still little attention to the possible reuse of treated water. For example, in the EU 40,000 million m^3^ of treated WW are produced every year, of which only 964 million m^3^ are reused [7]. Failure to recover the treated WW also involves developing countries where in many cases a significant plant shortage must also be addressed.

The EU adopted an ambitious Waste Circular Economy Package [24,25,26] in order to promote the reuse, recycling and recovery of raw materials to minimize the consumption of new resources [27,28]. WW can be considered a valuable resource that can help to overcome the water scarcity problem, especially in arid and semiarid climatic zones [29,30,31].

For WWs in recent years, the interest of research towards the recovery of greywater (GW) is becoming increasingly important, especially to cope with the scarcity of water at the domestic level [32,33,34,35]. As defined in literature, GW represents domestic WW generated from sinks, laundry washing and showers (excluding toilet flushing), and accounts for 50%–80% of the total domestic water use [5,36]. 

The GW has strong advantages as an alternative water source in arid and semiarid zones due to its continual supply, contrary to other supply methods such as rainwater harvesting, which depends on hydrological conditions, easy access in the household and a low level of contamination. This last feature allows removing contaminants with a simple treatment system chain, unlike other WW, making the onsite treatment of water possible [5]. In recent years, several types of processes have been tested on GW, such as filtration [37,38], membrane bioreactors [39,40], rotating biological contactors [35], sequencing batch reactor [41,42], up-flow anaerobic sludge blanket [43,44] and constructed wetlands (CW) [45,46].

Although there is no single optimal solution, which must be assessed according to a “case by case” logic, in recent years many studies have focused on the study of CW as a low impact system for the treatment of GW, especially in developing countries [47,48,49,50]. CW are complex systems that operate as biological filter hosting many abiotic and biotic processes capable of degrading or transforming many pollutants [45]. The main advantage of this system is linked to its low cost and the very low environmental impact, while among the most important disadvantages there is the low organic load that can be fed to the system which translates into high surfaces necessary to treat significant flow rates [51]. The CW can be classified according to the hydraulic flow scheme adopted: (i) vertical flow constructed wetlands (VFCW) and (ii) horizontal flow constructed wetlands (HFCW) [45].

In recent years, several studies have shown the applicability of HFCW as a treatment for the reuse of GW [52,53,54]. Different types of macrophytes have been tested and show good performance, such as *Cyperus papyrus*, *Cyperus iria, Typha latifolia*, *Phragmites australis* and bamboo plants [50,54,55,56]. Although there are some examples of applications on real waters [46,48,55], most of the research results described in the literature relate to tests conducted on synthetic waters.

In order to better simulate real conditions, this paper presents the results of more than four months of treatment of real GW by a HFCW. A significant aspect of this research is that a two-step approach was used. Firstly, a laboratory-scale HFCW was used to identify the performance of three macrophytes, *Carex oshimensis*, *Cyperus papyrus* and *Phragmites australis*, by testing them separately on the same GW. This represents an important aspect because in the literature no results have been presented regarding the use of *Carex oshimensis* for the treatment of GW. Secondly, pilot-scale tests were conducted to confirm the performance on a larger scale and to evaluate the influence of hydraulic retention time (HRT). Based on the results of the pilot-scale HFCW, a comparison with international recommendations by World Health Organization (WHO) and EU is discussed.

## 2. Materials and Methods 

### 2.1. Materials

#### 2.1.1. Greywater Collections

During the more than four month period of the study, GW was collected from different points of a house located in Italy: shower and sink of the bathroom, sink in the kitchen and from the washing machine.

#### 2.1.2. Laboratory-Scale Plant

**Figure 1:**
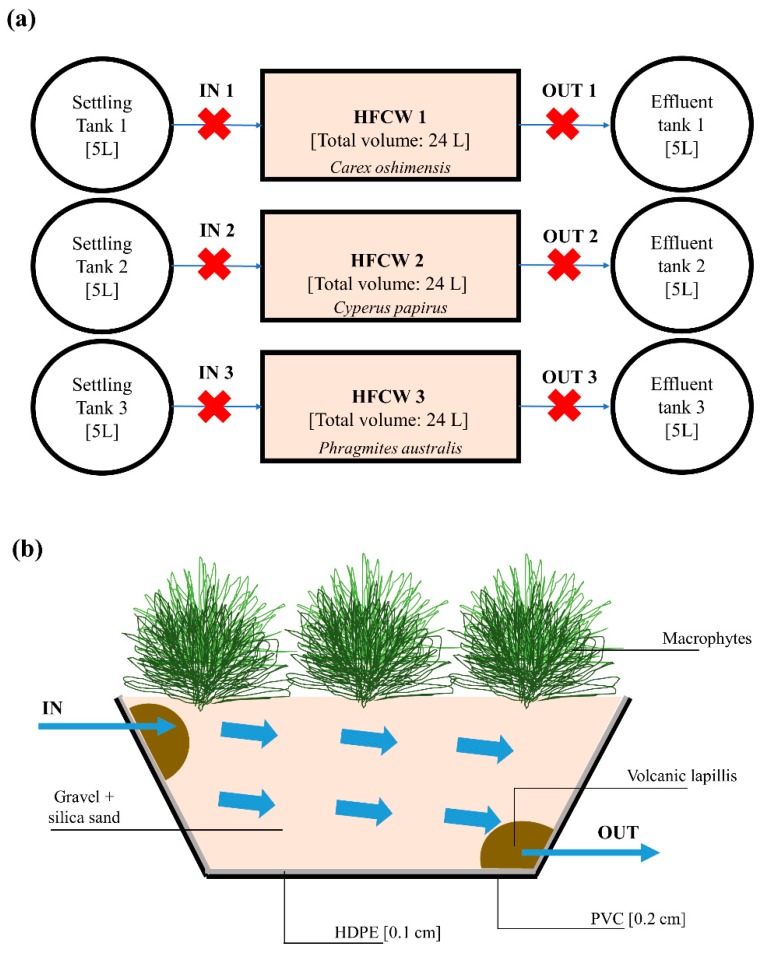
(**a**) Scheme of the preliminary laboratory-scale plant. The red crosses indicate the sampling points; (**b**) Side view of the horizontal flow constructed wetland (HFCW) laboratory-scale plant and pilot-scale plant. HDPE: high-density polyethylene, PVC: polyvinyl chloride.

A first preliminary laboratory-scale HFCW with submerged flow was realized in order to separately test the performance of three different types of macrophytes: *Carex oshimensis*, *Cyperus papyrus* and *Phragmites australis*. As shown in Figure 1a, the plant consisted of three parallel units, one for each type of macrophyte tested. To avoid obstructions of the connection pipes, sedimentation tanks (5 L) were located upstream from the respective HFCW in order to guarantee the sedimentation of the coarse material and the flotation of the oils. The HRT of the sedimentation tanks was 2 h.

Each HFCW unit (with dimensions 0.6 m× 0.2 m × 0.2 m) had a volume of 24 L and was planted with three macrophytes that after more than 1 month of the acclimatization phase covered the surface. The tanks of the units of HFCW were constructed of polyvinyl chloride (PVC) (Figure 1b). Each tank was further waterproofed with a layer of high-density polyethylene (HDPE) and subsequently filled with filter material (gravel and silica sand with a diameter of 2–10 mm) in order to obtain an effective porosity of the soil equal to 35%. The inlet and outlet pipes (1.1 cm internal diameter), made of polyethylene (PE), had 2 mm holes at regular distances. In order to ensure correct spreading of the inlet GW and correct collection of the treated GW at the outlet, a layer of draining material (volcanic lapilli with a diameter of 1–2 cm) was laid at the pipes. Both the inlet and outlet pipes were equipped with valves to control the incoming and outgoing flows. The average slope of the system was maintained at 0.5%–1.0%. The treated GW at the outlet was finally collected in three 5 L tanks.

The HFCW systems were illuminated by means of two lamps (15 W nominal power) for 12 h a day in order to promote chlorophyll photosynthesis.

#### 2.1.3. Pilot-Scale Plant

**Figure 2:**
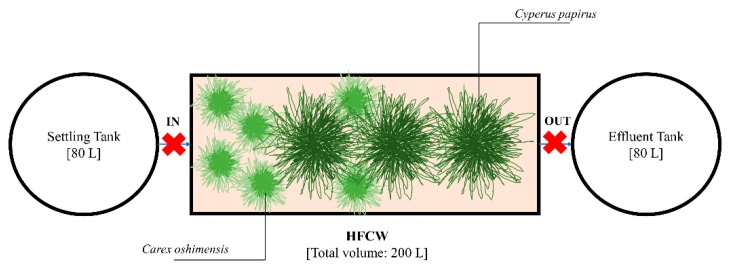
Schematic drawing of the pilot-scale plant. The red crosses indicate the sampling points. HFCW: Horizontal flow constructed wetland.

In order to test the CW on GW with the selected macrophytes (*Carex oshimensis* and *Cyperus papyrus*), a second larger pilot-scale plant HFCW with submerged flow was built (Figure 2). The structure of the treatment system and the illumination system were unchanged compared to the previous laboratory-scale plant (Figure 1b), but in this case the volumes were increased. The capacity of the tanks was 80 L and the HFCW, with dimension (0.8 × 0.6 × 0.4 m), had a volume of 200 L.

The position of the macrophytes was designed to ensure contact with GW (Figure 2). Considering that *Cyperus papyrus* are characterized by a root system capable of going deeper (up to 70 cm) [57,58], three specimens of this macrophyte were placed in the center towards the system outlet. As suggested by Masotti and Verlicchi [58], since the length of the roots of *Carex oshimensis* is limited (up to 10–30 cm) [59], six specimens of this macrophyte were positioned peripherally towards the tank entrance in order to ensure the contact with GW. After more than 1 month of acclimatization phase, the macrophytes covered the entire surface of the pilot-scale plant.

### 2.2. Methods

#### 2.2.1. Greywater Analysis

Five-day biological oxygen demand (BOD_5_), chemical oxygen demand (COD), total nitrogen (TN), nitrogen forms, total phosphorus (TP) and total suspended solids (TSS) concentrations were measured according to the Standard Methods for the Examination of Water and Wastewater [60]. For COD, TN, N-NO_2_^−^, N-NO_3_^−^ and TP analysis, Hach kits were used. Before the analysis, the interference from chlorides was ruled out by verifying that the chloride concentration was lower than the maximum accepted by the method.

Turbidity was measured using a WTW Turb 430 iR^®^ (Xylem, Germany) Dissolved oxygen (DO), pH and conductivity were measured using a portable multiparameter instrument (WTW 3410 SET4, Xylem, Germany). The DO was measured by using a WTW-IDS, model FDO^®^ 925 probe (Xylem, Germany). Electrical conductivity was measured using a WTW-IDS, model TetraCon^®^ 925 probe (Xylem, Germany). A WTW-IDS, Model SenTix^®^ 940 probe (Xylem, Germany) was used to measure pH. 

Water samples (300 mL) were collected before water entered and after it left the HFCW units (Figure 1a and Figure 2). *Escherichia coli* was determined by a membrane filtration technique (with a cellulose nitrate filter of 0.45 μm pore size, Millipore) and using a selective chromocult agar (NPS-COLC) culture medium [61,62].

#### 2.2.2. Plants Management and Monitoring

The HRT of the preliminary laboratory-scale HFCW was imposed and equal to 2 days. In order to study the influence of this parameter on the performance of the system, two HRT were tested (1 and 3 days) in the pilot-scale HFCW. As suggested by the Environmental Protection Agency [63], the optimal flowrate (Q) was calculated using Equation (1):

Q [L d^−1^] = n·V·HRT^−1^. (1)

where n represents the porosity of the system and V represents the bed volume. Considering the characteristics of the plants, a flowrate of 4 L d^−^^1^ was used in the preliminary laboratory-scale reactor, and flowrates of 23 L d^−^^1^ and 70 L d^−^^1^ were used in the pilot-scale plant, depending on the HRT.

The study consisted of two phases: (i) a preliminary laboratory-scale phase in order to test the performance of *Carex oshimensis*, *Cyperus papyrus* and *Phragmites australis* to treat GW with HFCW and (ii) a subsequent pilot-scale phase, with the aim of evaluating the influence of HRT and the treatment efficiency during a longer period. The first preliminary phase was conducted in various 2 days intervals over a 7 week period, and samples were collected on every second day. The second phase was conducted in various 1 and 3 days intervals over a 7 week period, depending on the HRT imposed. In both phases, the temperature in the room in which the HFCW operated was maintained at around 25 °C.

## 3. Results and Discussions

### 3.1. Laboratory-Scale Plant

In this first phase, a laboratory-scale HFCW was used to separately test the performance of three different types of macrophytes (*Carex oshimensis, Cyperus papyrus and Phragmites australis*). The influent and the effluent pH, conductivity, turbidity and concentration of COD, BOD_5_, TN and TP are shown in Table 1.

The pH was unchanged compared to that of the influent GW and this result was noted for all types of macrophytes studied. Moreover, the pH ranges remained within the recommended range for the existence of the majority of treatment bacteria (4.0 < pH < 9.5) [47], denitrifiers (7.5 < pH < 9.0) [64], and nitrifiers (7.5 < pH < 8.0) [65]. Compared to the influential GW, the conductivity significantly increased in all three units with the highest maximum values noted in the unit cultivated with *Cyperus papyrus.* This increase was probably due to the dissolution of some salt components present in the soil of the HFCW at the passage of the GW to be treated.

**Figure 3:**
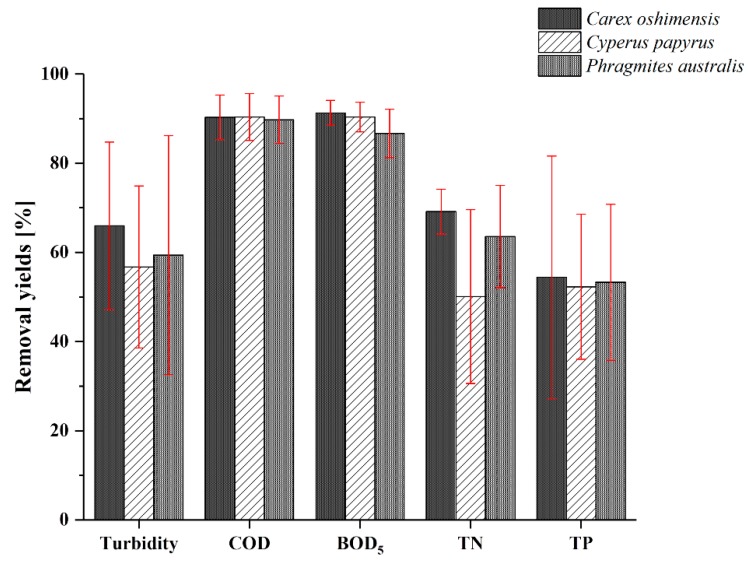
Removal yields of main chemical and chemical–physical contaminants by *Carex oshimensis*, *Cyperus papyrus* and *Phragmites australis*. The red bars indicate the 95% confidence intervals.

Furthermore, from the results obtained it is possible to highlight that all three types of plants showed significant removal yields on all the other chemical and chemical–physical parameters analysed (Figure 3). In terms of mean value, *Carex oshimensis* had the highest removal yields of turbidity (66%), BOD_5_ (91%), TN (69%) and TP (54%). COD was removed in equal percentage (90%) from the three plants studied. *Cyperus papyrus* also showed good results on BOD_5_ removal (90%). *Phragmites australis* showed results comparable to the other two types of plants, but slightly lower in terms of mean value than those obtained with the *Carex oshimensis* in turbidity (59%), BOD_5_ (87%) and TN (64%) removal.

Therefore, the performance of the three macrophytes on the removal of each single pollutant was very similar and there were no significant statistical differences (*p*-value was always higher than 0.05). The good results for *Carex oshimensis* were not obvious; in fact, literature on the application of this macrophyte for the treatment of real GW is absent. *Carex oshimensis* and *Cyperus papyrus* were chosen for combined use in the pilot-scale implant for several reasons:

(i) Test alternative solutions to well-known *Phragmites australis* and obtain other data on *Carex oshimensis* application on real GW treatment.
(ii) Nema et al. [50] highlighted that Phragmites australis showed good results on GW treatment in the short-term, but the same high performance cannot be maintained for a longer time without periods of rest. The pilot-scale implant overcomes this aspect.
(iii) One of the limits of *Carex oshimensis* is certainly linked to the shallow depth that its roots reach (maximum 10–30 cm) [59]. Therefore, the combination of *Carex oshimensis* and *Cyperus papyrus* guarantee to overcome this limit positioning *Carex oshimensis* in the part closest to the entrance of the GW, and locating *Cyperus papyrus* (characterized by a deeper root system) in the central part of the pilot-scale plant (For further details, please see Section 2.1).

### 3.2. Pilot-Scale Plant

The aim was to study the performance of *Carex oshimensis* and *Cyperus papyrus* at a larger scale and evaluate the influence of HRT. Two different phases were conducted: (i) phase P1 with HRT equals to 1 day and (ii) phase P2 with HRT of 3 days. Given the purposes, in addition to the parameters already monitored in the previous phase, a series of additional parameters were also monitored, namely TSS, N-NO_2_^−^, N-NO_3_^−^, total Kjeldahl nitrogen (TKN) and *Escherichia coli*.

Concerning pH, in both cases the results confirmed what was already highlighted in the laboratory phase of the study: a substantially unchanged situation between influent and treated GW (Table 2). Conductivity increased considerably with the 3 days HRT. The increase was probably due to the dissolution of saline components present in the soil. In fact, by decreasing the contact time in the system (HRT = 1 day), the dissolution of the saline components was not detected.

Table 2 shows similar results for turbidity, COD and BOD_5_ in both cases. Compared to the laboratory phase, much more significant removal yields of the turbidity (>92%) were obtained in the pilot-scale HFCW compared to the laboratory-scale plant. Excluding the influence of HRT (removal yields changing HRT were very similar), this result could be related to the combination of the two different macrophytes. The removal data of COD, BOD_5_ and TP obtained in the laboratory-scale reactor were fully confirmed.

The excellent removal yield of TSS and *Escherichia coli* can be highlighted. These results were also confirmed by other literature studies that highlighted the significant effect of CW in reducing health risk [45,49].

By increasing the HRT from 1 to 3 days, there seemed to be no substantial improvement in the performance of the HFCW except for the increase from 50.7% to 54.6% of the TP removal.

Regarding the TN, a higher removal (77.6%) was observed with HRT equal to 1 day (phase P1) compared to 42.5% obtained with a HRT of 3 days (phase P2). This was in contrast with literature reporting an increase in HRT produced higher removals of TN [66].

**Figure 4:**
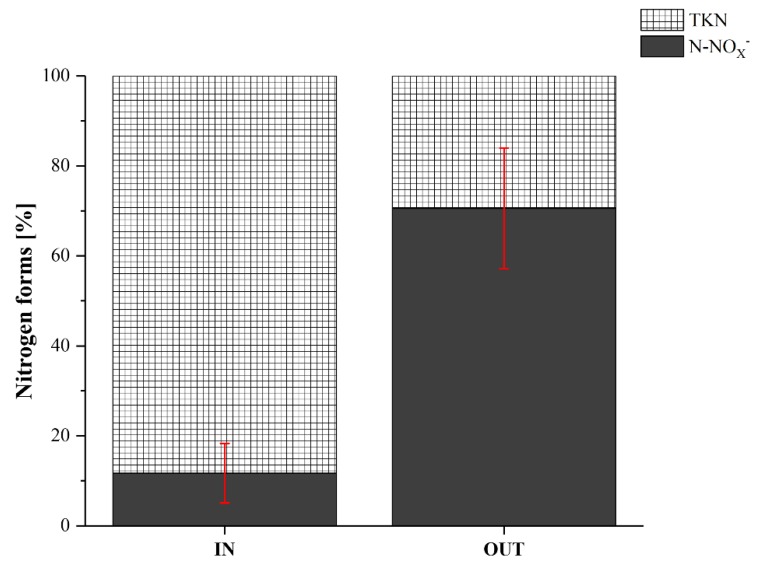
The components of the TN as a percentage by comparing the concentration of the influent GW and of the HFCW effluent in the phase P2 (HRT = 3 days). The red bars indicate the 95% confidence intervals.

This result could be related with to incomplete denitrification in phase P2. The larger part of TN in the influent GW was composed of N-NH_4_^+^ and N_org_ (Figure 4). The ability of *Carex oshimensis* and *Cyperus papyrus* to oxygenate the rhizosphere creating an area useful for the growth of nitrifying aerobic bacteria [67,68] allowed the transformation of ammonia in nitrates. Furthermore, the pH of the influent GW and of the effluent was always within the optimal range for the development and growth of nitrifying bacteria (Table 2). By monitoring the DO (Dissolved Oxygen) in the GW entering and leaving the filter, a slight decrease was found (5.16 to 4.06 mg L^−1^, on average). This decrease could be associated with the large demand of oxygen by the bacteria for degradation of organic substances and for nitrification, which therefore employ not only the oxygen introduced by the roots in the rhizosphere, but also the DO present in the GW. However, the nitrates remained in the system due to the absence of anoxic conditions or, more likely, to the low availability of organic matter available to denitrifiers. In fact, the average concentrations of COD and BOD_5_ in the influential GW of phase P2 were, respectively, 15% and 51% lower than in phase P1.

Another aspect that can be observed from the results was that the concentration of pollutants in the effluent had a much lower variability than the inlet concentrations (e.g., turbidity). This result was already detectable in the first phase of the experimentation and, given the larger dimensions of the HFCW, it was more evident both in phase P1 and in phase P2.

### 3.3. Comparison with Regulatory Parameters for Reuse

At an international level, the legislative framework on the reuse of GW after treatment is still very heterogeneous. Few countries have adopted legislation, including emerging ones where the treatment and recovery of these waters could represent a significant opportunity. Internationally, the WHO issued recommendations prescribing minimum requirements that the legislation should guarantee according to the final reuse of the purified GW [69]. However, these recommendations concern only a limited number of parameters (TSS, BOD_5_ and microbiological parameters) (Table 3). In order to standardize the regulatory framework, which is at times completely absent in some member countries, the EU also issued guidelines dividing the minimum requirements based on the type of reuse (agricultural, industrial, energy) [70].

By comparing the concentrations of the parameters analysed with those recommended by WHO, it can be stated that the GW treated with *Carex oshimensis* and *Cyperus papyrus* met the acceptance requirements for the irrigation of ornamental fruit trees and fodder crops. The BOD_5_ and microbiological parameter represented the critical values that prevented the reuse of the effluent of phase P1 and P2, respectively, for irrigation of vegetables likely to be eaten uncooked. The effluent from phase P2 (HRT = 3 days) can be classified as a water of Class C according to EU recommendations. Therefore, it can be used to irrigate (with drip irrigation only) food crops consumed raw where the edible portion is produced above ground and is not in direct contact with reclaimed water, processed food crops and non-food crops, including crops to feed milk- or meat-producing animals. Industrial reuse, energy reuse, and seeded crops irrigation are allowed, and in this case all irrigation methods are authorized.

Due to the critical value of BOD_5_, the treated effluent from phase P1 cannot be reused without further treatment.

Therefore, in order to have a treated GW quality capable of satisfying the most stringent requirements for potential urban reuse (e.g. toilet flushing) or for the irrigation of food crops where the edible portion is in direct contact with reclaimed water, it is necessary to intervene on the most critical parameters: BOD_5_ and the microbiological parameter. There could be two complementary solutions: (i) plan to couple the HFCW technology with a disinfection process for bacteria removal (preferably not using oxidizing agents such as Cl_2_ because it can form harmful disinfection by-products in presence of organic matter [71]), and (ii) ensure higher HRT (more than 3 days) in the system in order to improve the removal performance of BOD_5_.

## 4. Conclusions

In this study, a real GW was treated in order to test the performance of an HFCW. In the first phase *Carex oshimensis*, *Cyperus papyrus*, and *Phragmites australis* were separately tested in a laboratory-scale reactor in order to detect the performances of the three macrophytes. The results were very similar and *Carex oshimensis* and *Cyperus papyrus* were tested together on a pilot-scale reactor. The pilot-scale HFCW provided very high removal yields on turbidity (>92%), TSS (>85%), COD (>89%), and BOD_5_ (>88%). By increasing the HRT from 1 to 3 days, there appeared to be no substantial improvement in the performance of the HFCW except for the increase from 50.7% to 54.6% for the TP removal. A low removal of TN (42.5%) was observed in phase P2 when HRT equaled 3 days. It can be related to the low availability of organic matter in this phase, which led to incomplete denitrification. By comparing the results with the WHO and EU recommendations, it can be stated that in order to strengthen the quality of the treated GW, it is necessary to intervene on the most critical parameters: BOD_5_ and the microbiological parameter. There could be two complementary solutions: (i) plan to couple the HFCW technology with a disinfection process for bacteria removal, and (ii) ensure higher HRT (more than 3 days) in the system to improve the removal yield of BOD_5_.

## Figures and Tables

**Table 1:** Overall statistics of influent greywater (GW) and effluent concentrations in each unit.

		Influent GW Concentration ^1^	Effluent Concentration		
			Unit 1 *Carex oshimensis*	Unit 2 *Cyperus papyrus*	Unit 3 *Phragmites australis*
pH [–]	Mean	7.5	7.5	7.6	7.6
	C.I.	0.8	0.7	0.7	0.7
	Maximum value	8.5	8.4	8.5	8.5
	Minimum value	6.8	6.9	6.9	6.9
Electrical conductivity [μS cm^−1^]	Mean	481.3	809.3	823.0	733.7
	C.I.	59.3	146.6	250.9	165.4
	Maximum value	516	952	1079	891
	Minimum value	421	699	690	602
Turbidity [NTU]	Mean	121	30.8	41.3	36.2
	C.I.	59.4	6.6	7.5	12.7
	Maximum value	227	41.1	49.4	57.8
	Minimum value	64	22	29	21
COD [mg L^−1^]	Mean	1745.3	177.1	171.1	181.4
	C.I.	430.6	123.8	113.1	111.9
	Maximum value	2200	359	330	335
	Minimum value	1281	66	60	64
BOD_5_ [mg L^−1^]	Mean	720	65	70	107.5
	C.I.	153.7	5.7	17	70.3
	Maximum value	1000	70	95	215
	Minimum value	550	60	55	70
TN [mg L^−1^]	Mean	19.2	5.8	8.8	6.5
	C.I.	5	1.4	2.1	1
	Maximum value	24	8	12.2	7.6
	Minimum value	11.6	4	6.4	5.3
TP [mg L^−1^]	Mean	2.4	0.9	1	1
	C.I.	1.3	0.6	0.4	0.3
	Maximum value	5	2.1	1.6	1.4
	Minimum value	1.1	0.5	0.5	0.5

^1^ The values refer to the samples taken after the settling phase and immediately before the HFCW (see in detail the sampling points in Figure 1a). C.I.: confidence interval; BOD: biological oxygen demand; COD: chemical oxygen demand; TN: total nitrogen; TP: total phosphorus.

**Table 2:** Overall statistics of influent GW and effluent concentrations in the pilot-scale plant (phase P1 and P2) and removal yields of pollutants.

Parameter	Phase P1 [HRT = 1 day]							Phase P2 [HRT = 3 days]						
	Influent GW Concentration			Effluent Concentration			Removal Yields Phase P1 [% ± C.I.] ^1^	Influent GW Concentration			Effluent Concentration			Removal Yields Phase P2 [% ± C.I.] ^1^
	Mean Value [± C.I.]	Maximum Value	Minimum Value	Mean Value [± C.I.]	Maximum Value	Minimum Value		Mean Value [± C.I.]	Maximum Value	Minimum Value	Mean Value [± C.I.]	Maximum Value	Minimum Value	
pH [-]	8.3 [± 0.5]	8.8	7.5	8.0 [± 0.5]	8.7	7.2	[-]	8.3 [± 0.3]	8.9	7.7	8.0 [± 0.3]	8.5	7.3	[-]
Electrical conductivity [μS/cm]	1824.6 [± 683.9]	3580	531	1293.8 [± 191]	1884	783	[-]	401.9 [± 52.7]	561	356	1071.3 [± 140.8]	1377	890	[-]
Turbidity [NTU]	468.6 [± 157.7]	773	99	11.9 [± 5.1]	32	3	95.7 [± 2.8]	66.2 [± 26.3]	127	31	5.2 [± 3.1]	12	2.1	92 [± 3.9]
TSS [mg L^−1^]	27 [± 9.3]	44	4	1.5 [± 1.5]	5	0	96.3 [± 3.8]	49.7 [± 17.1]	80	20	8 [± 3.7]	16	0	84.6 [± 7.2]
COD [mg L^−1^]	1327.7 [± 275.8]	1794	469	122.6 [± 32.8]	221	66	89.5 [± 3.9]	1119 [± 350.8]	1900	494	119 [± 26]	172	62	88.8 [± 1.4]
BOD_5_ [mg L^−1^]	522.2 [± 94.4]	686	280	55 [± 9.6]	74	26	88.1 [± 3.7]	255.7 [± 33.9]	340	210	20.6 [± 5.5]	30	10	92 [± 2]
TN [mg L^−1^]	33.6 [± 8.2]	47.2	16.7	7.2 [± 2.6]	10.8	2.8	77.6 [± 8.7]	11 [± 2.9]	17	7.9	6.1 [± 1.1]	8	4.7	42.5 [± 11.1]
N-NO_2_^−^ [mg L^−1^]	n.a.	n.a.	n.a.	n.a.	n.a.	n.a.	n.a.	0.3 [± 0.3]	0.7	0.1	0.1 [± 0.1]	0.4	0.1	increase
N-NO_3_^−^ [mg L^−1^]	n.a.	n.a.	n.a.	n.a.	n.a.	n.a.	n.a.	0.9 [± 0.5]	1.3	0.1	3.8 [± 1.7]	4.9	1.3	increase
TKN[mg L^−1^]	n.a.	n.a.	n.a.	n.a.	n.a.	n.a.	n.a.	10.3 [± 4.9]	15.5	3.6	1.9 [± 1.4]	3.1	0	66.2 [± 14.5]
TP [mg L^−1^]	5.8 [± 2.7]	13	2.2	2.1 [± 0.4]	3	0.8	50.7 [± 19.5]	2.3 [± 0.6]	3.1	1.6	1 [± 0.2]	1.4	0.8	54.6 [± 7.8]
*Escherichia coli* [UFC 100^−1^ mL^−1^]	3.3 × 10^4^ [± 1 × 10^4^]	5.0 × 10^4^	1.8 × 10^2^	17.2 [± 13.5]	60	1	2.8 log [± 0.8 log]	2.4 × 10^5^ [± 1.7 × 10^5^]	4 × 10^5^	1.1 × 10^5^	937 [± 361]	1.3 × 10^3^	7 × 10^2^	2.4 log [± 0.2 log]

^1^ The removal yields of *Escherichia coli* are expressed in log scale and not as a percentage. C.I.: confidence interval; TKN: total Kjeldahl nitrogen; n.a.: not available; HRT: hydraulic retention time; TSS: total suspended solids.

**Table 3:** Comparison between the characteristics of the effluent and the international recommendations for the reuse of treated GW. TKN: total Kjeldahl nitrogen; n.a.: not available; n.p.: not provided.

Parameters	Pilot-Scale HFCW Effluent [Phase PA]	Pilot-Scale HFCW Effluent [Phase PB]	WHO Recommendations [69]			EU Recommendations [70]			
			Irrigation of Ornamental Fruit Trees and Fodder Crops	Irrigation of Vegetables Likely to be Eaten Uncooked	Toilet Flushing	Class A ^1^	Class B ^2^	Class C ^3^	Class D ^4^
pH [-]	8.0 ± 0.5	8 ± 0.3	n.p.	n.p.	n.p.	n.p.	n.p.	n.p.	n.p.
Conductivity [μS cm^−1^]	1293.8 ± 191	1071 ± 141	n.p.	n.p.	n.p.	n.p.	n.p.	n.p.	n.p.
Turbidity [NTU]	11.9 ± 5.1	5.2 ± 3.1	n.p.	n.p.	n.p.	5	n.p.	n.p.	n.p.
TSS [mg L^−1^]	1.5 ± 1.5	8 ± 3.7	140	20	10	10	35	35	35
COD [mg L^−1^]	122.6 ± 32.8	119 ± 26	n.p.	n.p.	n.p.	n.p.	n.p.	n.p.	n.p.
BOD_5_ [mg L^−1^]	55 ± 9.6	20.6 ± 5.5	240	20	10	10	25	25	25
TN [mg L^−1^]	7.2 ± 2.6	6.1 ± 1.1	n.p.	n.p.	n.p.	n.p.	n.p.	n.p.	n.p.
N-NO_2_^−^ [mg L^−1^]	n.a.	0.1 ± 0.1	n.p.	n.p.	n.p.	n.p.	n.p.	n.p.	n.p.
N-NO_3_^−^ [mg L^−1^]	n.a.	3.8 ± 1.7	n.p.	n.p.	n.p.	n.p.	n.p.	n.p.	n.p.
TKN [mg L^−1^]	n.a.	1.9 ± 1.4	n.p.	n.p.	n.p.	n.p.	n.p.	n.p.	n.p.
TP [mg L^−1^]	2.1 ± 0.4	1 ± 0.3	n.p.	n.p.	n.p.	n.p.	n.p.	n.p.	n.p.
*Escherichia coli* [UFC 100^−1^ mL^−1^]	17.2 ± 13.5	937 ± 237	1000 ^5^	200 ^5^	10 ^5^	10	100	1000	1000

^1^ Minimum reclaimed water quality for all food crops, including root crops consumed raw and food crops where the edible portion is in direct contact with reclaimed water (all irrigation methods allowed); ^2^ Minimum reclaimed water quality for food crops consumed raw where the edible portion is produced above ground and is not in direct contact with reclaimed water, processed food crops and nonfood crops, including crops to feed milk- or meat-producing animals (all irrigation methods allowed); ^3^ Minimum reclaimed water quality for food crops consumed raw where the edible portion is produced above ground and is not in direct contact with reclaimed water, processed food crops and nonfood crops including crops to feed milk- or meat-producing animals (drip irrigation only); ^4^ Minimum reclaimed water quality for industrial reuse, energy reuse, and seeded crops (all irrigation methods allowed); ^5^ This value is referred to the total number of thermotolerant coliforms.

## Nomenclature

BOD	Biochemical oxygen demand
COD	Chemical oxygen demand
CW	Constructed wetland
DO	Dissolved oxygen
EEA	European Environmental Agency
EU	European Union
GW	Greywater
HDPE	High-density polyethylene
HFCW	Horizontal flow constructed wetland
HRT	Hydraulic retention time
Phase P1	Phase of pilot-scale reactor with HRT equals to 1 day
Phase P2	Phase of pilot-scale reactor with HRT equals to 3 days
PVC	Polyvinyl chloride
TKN	Total Kjeldahl nitrogen
TN	Total nitrogen
TP	Total phosphorus
TSS	Total suspended solids
VFCW	Vertical flow constructed wetlands
WEI	Water exploitation index
WHO	World Health Organization
WW	Wastewater
